# Book Review: Kiknadze I., Istomina A., Golygina V., Gunderina L. Karyotypes of Palearctic and Holarctic species of the genus *Chironomus* [Electronic resource] Russian Academy of Sciences, Siberian Branch, Federal Research Center, Institute of Cytology and Genetics. Novosibirsk: Academic Publishing House “GEO”, 2016. – 489 p. ISBN 978-5-9908853-2-5.

**DOI:** 10.3897/CompCytogen.v11i2.13527

**Published:** 2017-06-19

**Authors:** Valentina Kuznetsova

**Affiliations:** 1 Department of Karyosystematics, Zoological Institute, Russian Academy of Sciences, Universitetskaya nab. 1, 199034, St. Petersburg, Russia

**Figure 1.**
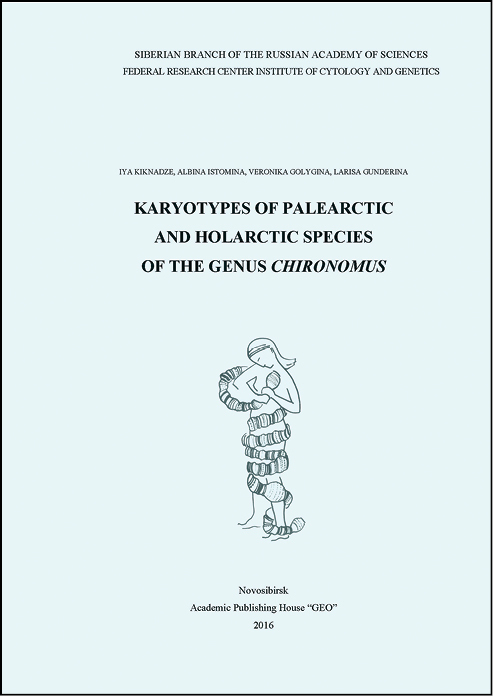
Fig

The book “Karyotypes of Palearctic and Holarctic species of the genus *Chironomus*” was published last year in the Russian Federation by Ija Kiknadze, Albina Istomina, Veronika Golygina and Larissa Gunderina (Fig. 1).

Professor I. Kiknadze and her co-authors are noted scientists, who have had a long academic experience in comparative cytogenetics and karyosystematics of the family Chironomidae (Insecta, Diptera, Nematocera), primarily of the species-rich and taxonomically complicated genus *Chironomus* Meigen, 1803, and have made a significant contribution to our understanding of the cytogenetic mechanisms of microevolution in natural systems.

The genus *Chironomus* includes several hundred species distributed almost worldwide (except Antarctica). *Chironomus* larvae are of great concern in aquatic ecosystems and are widely used in ecological and environmental studies of fresh waters. The species identification of *Chironomus* larvae is hence of considerable importance. It is, however, difficult due to the small number of distinct morphological differences between the larvae of many species, especially between those belonging to the sibling species groups. It is generally agreed that the definitive identification of the larvae can be performed using a combination of different lines of evidence including the analysis of traditional morphological characters, molecular markers and structure of salivary-gland polytene chromosomes.

The book is a tribute to the enormous amount of cytogenetic research on the genus *Chironomus* that has been done by the authors during the decades. The book begins with the table of contents, list of abbreviations and acronyms used to denote the transcriptionally active regions of chromosomes, geographic ranges of chromosome banding sequences, banding frequency occurrence and some other terms, and the list of the studied *Chironomus* sibling species groups (the *aberratus*, the *obtusidens*, the *piger*, the *plumosus* andthe *riihimakiensis* groups).

The main body of the book is separated in the two chapters. Chapter I provides the reader with exhaustive and up-to-date information on karyotype structure, polytene chromosome banding sequences and inversion polymorphism in *Chironomus*. Numerous visual displays such as drawings, diagrams, photos, tables, and schemes help the understanding of these complex data.

Michael White, in his notable book “Modes of speciation” (1978) states that “over 90 percent of all speciation events are accompanied by karyotypic changes and in the majority of these cases the structural chromosomal rearrangements have played a primary role in initiating divergence” (p. 324). The book of Kiknadze with co-authors demonstrates well that *Chironomus* species, given the ease of analyzing their giant polytene chromosomes, are the unique subjects for studying the microevolution processes, i.e. evolution on a small-scale. The reader is introduced to the authors’ findings making it apparent that most cases of chromosomal polymorphism in *Chironomus* are represented by homo- and heterozygous paracentric inversions. Besides, rare heterozygotes for pericentric inversions, reciprocal translocations and size variation of homologous bands including the centromeric bands (band thickness) are known. Polymorphism for B chromosomes is shown to occur as well.

Chapter II covers the original authors’ data and addresses the karyotypes (more precisely, the chromosomal polymorphisms) of sixty three *Chironomus* species referred to the five groups of sibling species and originated from different regions including Russia (European part, Ural, West and East Siberia, Altai, Tuva, the Far East), Kazakhstan, West Europe (Germany, Belgium, the Netherlands, Bulgaria), USA, Canada, China, and Japan. The chapter is particularly noteworthy because of the detailed and fully illustrated presentation of chromosomal polymorphisms in each species and each population under study. What I liked most in this chapter were the wonderful pictures of polytene chromosomes. The polymorphisms are considered and discussed in the context of evolutionary divergence of species and populations and are used as a basis for reconstruction of ancestral chromosome architecture and further chromosome evolution in the genus *Chironomus*. The banding sequence pattern in *Ch. piger* Strenzke, 1959 suggested by Keyl (1962) as a standard one is applied for chromosome mapping in the species under study.

The book is completed with an extensive reference list, which comprises over 250 items and makes this an indispensable work for every student in *Chironomus* cytogenetics and systematics.

I expect this book to be very well accepted by entomologists, cytogeneticists, evolutionary biologists and advanced students doing research on chironomid cytology, ecology and systematics. The ability to accurately identify *Chironomus* species at the larva stage should facilitate taxonomic, ecological and environmental studies of the group in different geographical regions. Proulx et al. (2013) are still of the opinion (and this is my opinion as well) that only a few of the taxonomists worldwide display the necessary expertise to identify *Chironomus* species using chromosome structure, which is a major drawback for non-cytological experts wishing to do it. In this context, my only criticism of the book by Kiknadze with co-authors from a student’s perspective is the conspicuous absence of a glossary. Such a glossary would help the students in defining numerous and highly specific terms that appear throughout the text.

Despite this criticism, the book is excellent, and I would recommend it to everyone who is interested in the genus *Chironomus* cytogenetics and systematics.

The production of the book was carried out with the financial support of the budgetary project of the Russian Academy of Sciences (№ 0324-2015-0003).
